# Unveiling Conformational States of CDK6 Caused by Binding of Vcyclin Protein and Inhibitor by Combining Gaussian Accelerated Molecular Dynamics and Deep Learning

**DOI:** 10.3390/molecules29112681

**Published:** 2024-06-05

**Authors:** Lu Zhao, Jian Wang, Wanchun Yang, Kunpeng Zhao, Qingtao Sun, Jianzhong Chen

**Affiliations:** School of Science, Shandong Jiaotong University, Jinan 250357, China; wangjian_lxy@sdjtu.edu.cn (J.W.); yangwch1982@126.com (W.Y.); 204085@sdjtu.edu.cn (K.Z.); 211103@sdjtu.edu.cn (Q.S.)

**Keywords:** CDK6, gaussian accelerated dynamics simulations, deep learning, free energy landscape

## Abstract

CDK6 plays a key role in the regulation of the cell cycle and is considered a crucial target for cancer therapy. In this work, conformational transitions of CDK6 were identified by using Gaussian accelerated molecular dynamics (GaMD), deep learning (DL), and free energy landscapes (FELs). DL finds that the binding pocket as well as the T-loop binding to the Vcyclin protein are involved in obvious differences of conformation contacts. This result suggests that the binding pocket of inhibitors (LQQ and AP9) and the binding interface of CDK6 to the Vcyclin protein play a key role in the function of CDK6. The analyses of FELs reveal that the binding pocket and the T-loop of CDK6 have disordered states. The results from principal component analysis (PCA) indicate that the binding of the Vcyclin protein affects the fluctuation behavior of the T-loop in CDK6. Our QM/MM-GBSA calculations suggest that the binding ability of LQQ to CDK6 is stronger than AP9 with or without the binding of the Vcyclin protein. Interaction networks of inhibitors with CDK6 were analyzed and the results reveal that LQQ contributes more hydrogen binding interactions (HBIs) and hot interaction spots with CDK6. In addition, the binding pocket endures flexibility changes from opening to closing states and the Vcyclin protein plays an important role in the stabilizing conformation of the T-loop. We anticipate that this work could provide useful information for further understanding the function of CDK6 and developing new promising inhibitors targeting CDK6.

## 1. Introduction

Cyclin-dependent kinases (CDKs) are a class of protein kinases that play a key role in the regulation of the cell cycle [1,2,3,4,5,6,7,8,9,10]. According to their respective functions, CDKs can be categorized into two major groups: cell cycle-regulated CDKs (such as CDK1, CDK2, CDK4, and CDK6) and transcription-regulated CDKs (such as CDK7, CDK8, and CDK9) [11,12]. Throughout the progression of the cell cycle, these CDKs sequentially regulate various mitotic events. Under the influence of diverse growth signals, phosphorylation of pRb is initiated by CDK4–Cyclin D and CDK6–Cyclin D, thereby initiating the cell cycle. Subsequently, the CDK2–Cyclin E complex keeps pRb highly phosphorylated, regulating the entry of cells into the S phase and centrosome replication. CDK1 is a key determinant of mitosis and forms active complexes with Cyclin A and B in the latter half of the G2 phase and throughout the M phase, respectively. Furthermore, CDK5 plays a crucial role in the regulation of post-mitotic events in specific tissues [11]. In cancer cells, CDKs lead to abnormal cell proliferation due to their over-activation [13,14]. Consequently, CDKs have been regarded as a potential key target for cancer therapy [15,16,17]. Exploring the molecular-level structures of CDKs is imperative for developing next-generation CDK inhibitors. However, much of the current research is primarily focused on CDK2, with limited investigation into CDK6.

**Figure 1:**
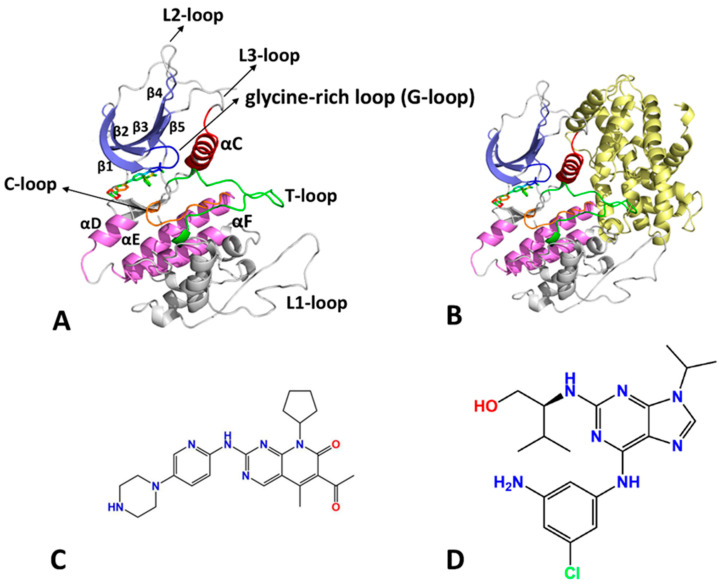
Molecular structure: (**A**) the LQQ-bound CDK6, the main components of catalytic pocket are shown in blue (β-strands), red (αC-helix), green (T-loop), orange (C-loop), and violet (C-lobe helix), (**B**) the LQQ-bound CDK6/Vcyclin complex. Vcyclin is shown in yellow, (**C**,**D**) correspond to LQQ and AP9, in which two inhibitors are displayed in line form.

CDK6, a pivotal member of the CDK family, integrates mitogenic and anti-mitogenic extracellular signals with the cell cycle, exerting significant regulation during the G1 phase. CDK6 has similar structures to other CDK members, characterized by an N-lobe domain comprising five β-strands, a C-lobe domain consisting of multiple α-helices, and the catalytic domain featuring as an interconnecting region between the N-lobe and C-lobe domains [18], as depicted in Figure 1A. The substrates of CDK6, including ATP and peptides, bind to the catalytic sites of CDK6, initiating a catalytic reaction that leads to the phosphorylation of peptides as well as the production of ADP and H_2_O. The catalytic domain of CDK6 primarily encompasses an ATP binding pocket, a phosphate transfer catalytic loop (C-loop: residue: 141–151), and an activation loop (T-loop: residue: 163–184) responsible for substrate peptide binding. CDK6 activity is finely regulated by its binding to cyclin protein and subsequent phosphorylation on the T-loop [19,20,21,22,23,24,25,26,27,28,29,30].

In the CDK6–Vcyclin complex, a contiguous protein–protein interface was established by the interaction of Vcyclin with one side of the CDK6 catalytic domain, as depicted in Figure 1B. For the αC helix, the Vcyclin protein drives it to shift and rotate toward the catalytic domain. As a result, the E61 residue of CDK6 on the αC helix is oriented toward the ATP binding pocket, thereby contributing to stabilization of the ATP binding conformation. For the T-loop, five hydrogen bonds and a large number of van der Waals contacts between the Vcyclin and T-loop residues in CDK6 lock the T-loop in a stable conformation. V181 of CDK6 adopts the left-handed conformation, which is necessary to form a substrate binding pocket. Unlike CDK2, residues 171–174 in CDK6 adopt a classical type II turn conformation, contributing to the larger buried surface [3]. Therefore, it is highly requisite to probe the molecular mechanism underlying the conformational regulation of CDK6 caused by the binding of inhibitors and Vcyclin for the development of efficient inhibitors targeting CDK6.

Molecular dynamics (MD) simulation is a computational technique that provides valuable insights into biomolecular dynamics at atomic levels [31,32,33,34,35]. Meanwhile, calculations of binding free energies are used as a tool for evaluating the binding ability of inhibitors to targets [36,37,38]. These two simulation methods have been extensively applied in the conformation analysis of CDKs [39,40,41,42,43,44]. These studies suggest that CDK6 exhibits significant conformational flexibility and undergoes multiple conformations during the reaction cycle. Notably, the conformation of the catalytic domain is closely related to the action mechanism of CDK6 and binding to specific inhibitors. Conformations sampled by conventional MD (cMD) simulations are possibly trapped within an energy minima space due to the high energy barrier in simulation systems [45,46,47,48,49]. To address this limitation, Gaussian accelerated molecular dynamics (GaMD) simulations employ a harmonic boost potential to smooth the free energy barrier of biomolecules [50,51]. Moreover, GaMD ensures the accurate calculation of free energy profiles by minimizing energy noise during the reweighting process [52,53]. These attributes make GaMD particularly well-suited for the investigation of larger biological systems and ligand–target binding [54,55,56,57,58,59,60]. To better understand the molecular mechanism from MD simulations, the integration of machine learning (ML) with MD simulations has been proposed [61,62]. Miao’s group proposed a trajectory-based deep learning (DL) approach, called GaMD, DL, and free energy profiling workflow (GLOW), to successfully decipher the molecular mechanisms underlying the activation and allosteric modulation of G protein-coupled receptors [63,64]. Wang et al. combined MD simulations and DL to successfully probe the binding mechanism of inhibitors to BRD4 and BRD9 [65]. However, DL has not been combined with GaMD for the conformation analysis on inhibitor-bound CDK6.

To achieve our goal, four complexes, including the LQQ-bound CDK6/Vcyclin, LQQ-bound CDK6, AP9-bound CDK6/Vcyclin, and AP9-bound CDK6, were selected to investigate the influence of Vcyclin protein and inhibitor binding on CDK6 conformations. Two inhibitors, specific inhibitor LQQ, and less specific inhibitor AP9, indicated by using their identity document (ID) in the protein data bank (PDB), were selected for our current studies. The topological structures of the inhibitor–CDK6 complex and inhibitor–CDK6/Vcyclin are depicted in Figure 1A,B, respectively. The structures of LQQ and AP9 are separately displayed in Figure 1C,D. Two inhibitors, LQQ and AP9, have IC50 values of 15 and 450 nM, respectively, showing different inhibition abilities on the activity of CDK6 [66]. Insights into the effect of the two inhibitors and Vcyclin protein on the conformational changes of CDK6 will be important in the design of potent inhibitors. In this work, multiple separate GaMD (MS-GaMD) simulations were carried out to enhance the conformational sampling of CDK6, and DL was performed to identify key residue contacts in CDK6. Additionally, principal component analysis (PCA) [67,68,69,70] and construction of free energy landscapes (FELs) were performed to reveal the changes in the conformational dynamics of CDK6 induced by the binding of inhibitors and Vcyclin protein. We anticipate that this study will provide valuable information for the development of the potential inhibitor against CDK6.

## 2. Results and Discussion

### 2.1. Characteristic Residue Contacts Revealed by Deep Learning

**Figure 2:**
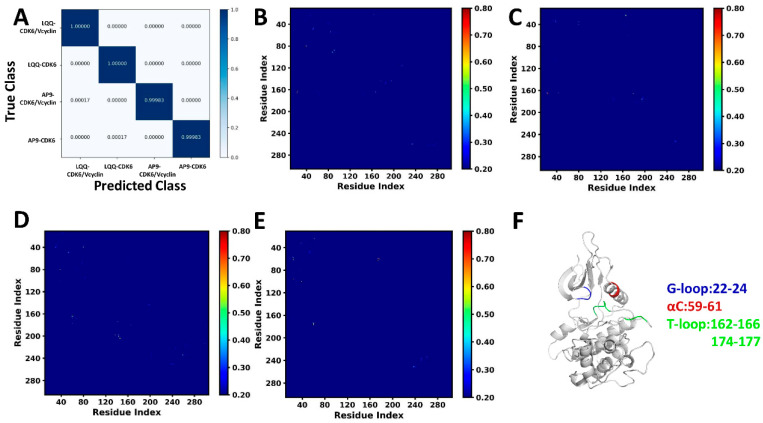
Classification and saliency map of residue contact gradients: (**A**) classification of the LQQ-bound CDK6/Vcyclin, LQQ-bound CDK6, AP9-bound CDK6/Vcyclin, and AP9-bound CDK6, (**B**–**E**) the saliency map of residue contact gradients for the LQQ-bound CDK6/Vcyclin, LQQ-bound CDK6, AP9-bound CDK6/Vcyclin, and AP9-bound CDK6, and (**F**) key structural domains revealed by DL. The gradient of each residue contact is shown in a 0.2 (blue) to 0.8 (red) color scale.

Classification of the LQQ-bound CDK6/Vcyclin, LQQ-bound CDK6, AP9-bound CDK6/Vcyclin, and AP9-bound CDK6 was achieved by DL, and the results are shown in Figure 2A. The overall accuracy achieved on the validation set after 25 epochs was 0.99992, while the overall loss was 0.00019 (Figure S1). In total, 6000 frames were used for the validation of each system with most of them being accurately classified (Figure 2A), including 6000 frames of the LQQ-bound CDK6/Vcyclin, 6000 frames of the LQQ-bound CDK6, 5999 frames of the AP9-bound CDK6/Vcyclin, and 5999 frames of the AP9-bound CDK6. Only two frames were inaccurately categorized: one frame of the AP9-bound CDK6/Vcyclin was predicted to be the LQQ-bound CDK6/ Vcyclin, and one frame of the AP9-bound CDK6 was predicted to be the LQQ-bound CDK6.

The pixel-based residue attention maps of gradients of the most populated CDK6 structures are shown in Figure 2B–E, respectively. The residue contacts with gradients ≥ 0.7 that are characteristic residue contacts are shown in Table S1. Overall, the characteristic residue contacts of the LQQ-bound CDK6/Vcyclin were located between the G-loop and T-loop (Table S1 and Figure 2F). The characteristic residue contacts of the LQQ-bound CDK6 were also located between the G-loop and T-loop (Table S1 and Figure 2F). The characteristic residue contacts of the AP9-bound CDK6/Vcyclin were situated between the αC helix and T-loop (Table S1 and Figure 2F). Compared to the LQQ-bound CDK6/Vcyclin and LQQ-bound CDK6, the AP9-bound CDK6/Vcyclin leads to the disappearance of the characteristic residue contacts between the G-loop and T-loop, and induces new characteristic residue contacts as we mentioned above. The characteristic residue contacts of the AP9-bound CDK6 were located between the αC helix and T-loop (Table S1 and Figure 2F). By referencing the AP9-bound CDK6/Vcyclin, the AP9-bound CDK6 changes the residue contact from the T-loop. In the AP9-bound CDK6/Vcyclin, the residues of the T-loop identified by DL were situated in the binding pocket; however, in the AP9-bound CDK6, the residue contacts from the T-loop were located near the binding interface between CDK6 and the Vcyclin protein.

Through the characteristic residue contacts learned by DL, the involved structure domains are primarily involved in the G-loop, the αC helix, and the T-loop. The work of Schulze-Gahmen et al. indicated that the G-loop, the αC helix, and the T-loop participate in the stable conformation of the binding pocket from CDK6 while the T-loop and the αC helix are heavily affected by the binding of Vcyclin [3], which agrees with our current findings learned by DL.

### 2.2. Conformational Transition of CDK6 from Free Energy Landscapes

**Figure 3:**
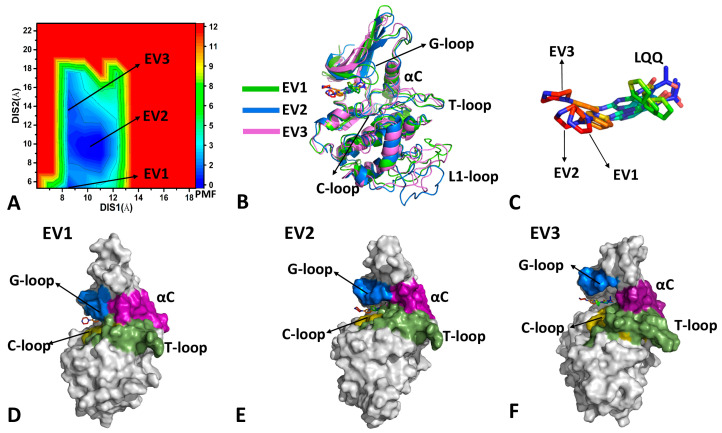
FEL constructed by using DSI1 and DIS2 as RCs and representative structures of the LQQ-bound CDK6/Vcyclin: (**A**) FEL, (**B**) superimposition of the EV1, the EV2, and the EV3 structures, (**C**) structural superimposition of LQQ in the EV1, the EV2, and the EV3 structures, (**D**–**F**) geometric positions of the G-loop, the αC helix, the T-loop, and the C-loop in the EV1, the EV2, and the EV3 structures, in which CDK6 was shown in surface modes. The PMF is scaled in kcal/mol.

**Figure 4:**
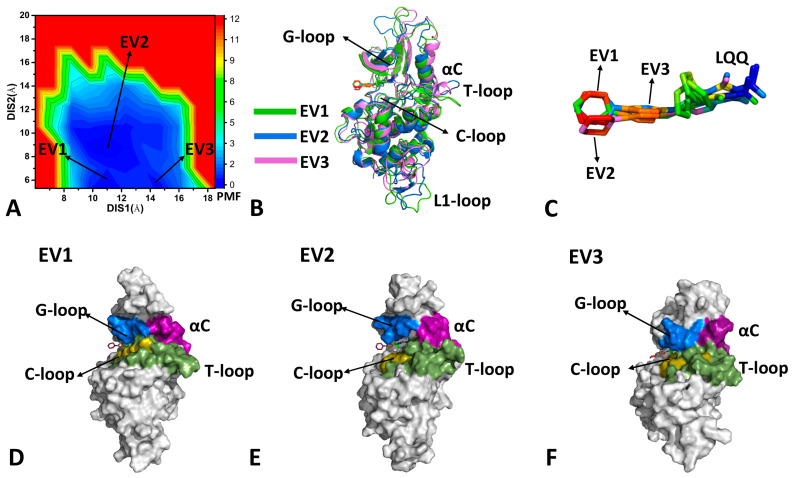
FEL constructed by using DSI1 and DIS2 as RCs and representative structures of the LQQ-bound CDK6: (**A**) FEL, (**B**) superimposition of the EV1, the EV2, and the EV3 structures, (**C**) structural superimposition of LQQ in the EV1, the EV2, and the EV3 structures, (**D**–**F**) geometric positions of the G-loop, the αC helix, the T-loop, and the C-loop in the EV1, the EV2, and the EV3 structures, in which CDK6 was shown in surface modes. The PMF is scaled in kcal/mol.

**Figure 5:**
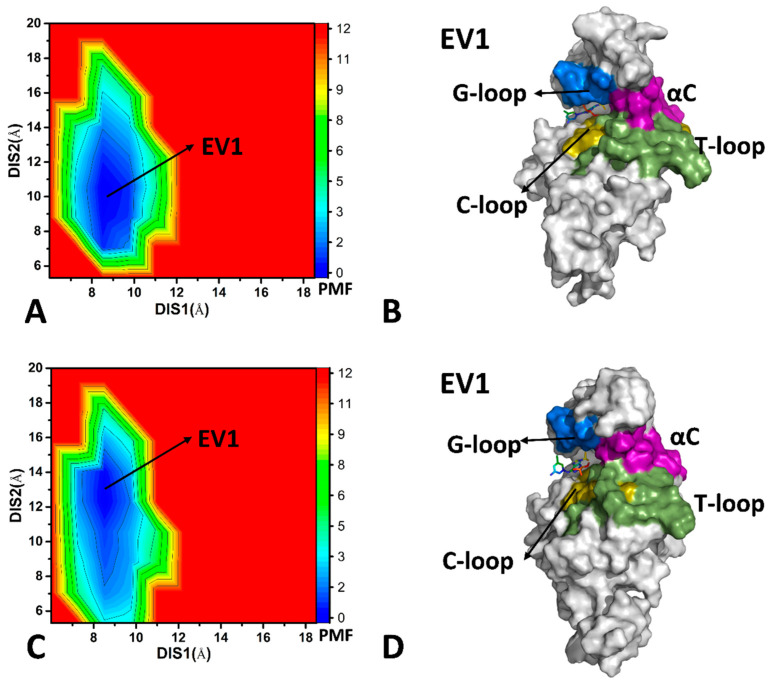
FEL constructed by using DSI1 and DIS2 as RCs and representative structures of the AP9-bound CDK6/Vcyclin and AP9-bound CDK6: (**A**) FEL of AP9-bound CDK6/Vcyclin, (**B**) geometric positions of the G-loop, the αC helix, the T-loop, and the C-loop in the EV1 structure of AP9-bound CDK6/Vcyclin, (**C**) FEL of AP9-bound CDK6, (**D**) geometric positions of the G-loop, the αC helix, the T-loop, and the C-loop in the EV1 structure of AP9-bound CDK6. The PMF is scaled in kcal/mol.

The previous DL analysis suggests that the residue contacts are mainly located near the binding pocket and binding interface between CDK6 and the Vcyclin protein. Residues located in the binding pocket identified from the DL results with the gradient of 0.9 were selected to calculate distances. Thus, we calculate the distance 1 (DIS1) between the Cα atom of residue E61 in the αC helix and the Cα atom of residue A162 in the T-loop and the distance 2 (DIS2) of the Cα atoms of residue G22 and residue A23 in the G-loop, respectively, away from the Cα atoms of residue D163 and residue L166 in the T-loop. The DIS1 and DIS2 were selected as the RCs to build FELs so as to reveal the binding pocket conformations of CDK6. The FELs and the corresponding representative structures are depicted in Figure 3, Figure 4 and Figure 5.

For the LQQ-bound CDK6/Vcyclin, three energy valleys (EV1-EV3) were identified by GaMD simulations (Figure 3A). EV1, EV2, and EV3 are, respectively, situated at the (DIS1, DIS2) of (8.6 Å, 6.0 Å), (10.5 Å, 9.6 Å), and (8.6 Å, 13.7 Å) (Figure 3A). The superimposition of the representative structures EV1-EV3 shows that the G-loop and the L1-loop have a highly disordered state, while the αC helix and T-loop have a relatively stable one (Figure 3B). The structural alignment of the LQQ in structures EV1, EV2, and EV3 indicates that some slight twists of the posture of the LQQ are captured (Figure 3C). The binding pocket of structure EV1 forms a closed state (Figure 3D), that of structure EV2 shows a semi-open one (Figure 3E), and that of structure EV3 has an open state (Figure 3F). Based on these results, the up–down moving of the G-loop results in the open and closed states of the binding pocket during GaMD simulations. Moreover, the conformational transformation between the open and closed states of the binding pocket induces the twist of the LQQ posture.

In the case of LQQ-bound CDK6, the conformational space expands significantly. Three energy valleys (EV1-EV3) were captured by GaMD simulations and they were located at the (DIS1, DIS2) of (10.9 Å, 5.9 Å), (11.1 Å, 8.9 Å), and (14.3 Å, 5.8 Å) (Figure 4A). The alignment of structures EV1-EV3 verifies that the G-loop, the αC, the T-loop, and the L1-loop possess highly disordered states (Figure 4B). Differently, the LQQ in structure EV3 produces a small twist relative to EV1, while that in structure EV2 produces a big twist (Figure 4C). The binding pocket of structure EV1 forms a closed state (Figure 4D), that of structure EV2 shows a semi-open one (Figure 4E), and that of structure EV3 has a closed state (Figure 4F). Compared to structure EV1, structure EV3 has a greater distance between the αC helix and the T-loop. By referencing to the LQQ-bound CDK6/Vcyclin, the LQQ-bound CDK6 has no open state. Moreover, the LQQ-bound CDK6 induces more disordered states of the αC helix and the T-loop. Thus, binding of the Vcyclin protein contributes to the stable conformation of the αC and the T-loop [3]. As a result, removing the Vcyclin protein leads to more disordered states of the αC helix and the T-loop.

With regard to the AP9-bound CDK6/Vcyclin and AP9-bound CDK6, only an energy valley (EV1) was captured by GaMD simulation, and it was situated at (DIS1, DIS2) of (8.6 Å, 10.1 Å) and (8.5 Å, 13.1 Å), respectively (Figure 5A,C), implying that the binding of AP9 and Vcyclin does not lead to conformational rearrangement of CDK6. The two FELs exhibit some similarity; however, the DIS2 in the AP9-bound CDK6 is higher than that of the AP9-bound CDK6/Vcyclin. The binding pocket of structure EV1 in the AP9-bound CDK6/Vcyclin forms a semi-open state, while that of structure EV1 in the AP9-bound CDK6 shows a more open one (Figure 5B,D). Compared with the LQQ-bound CDK6/Vcyclin and LQQ-bound CDK6, the AP9-CDK6/Vcyclin and AP9-CDK6 have no closed state of the binding pocket. Thus, different inhibitors certainly impact the binding pocket conformation of CDK6.

**Figure 6:**
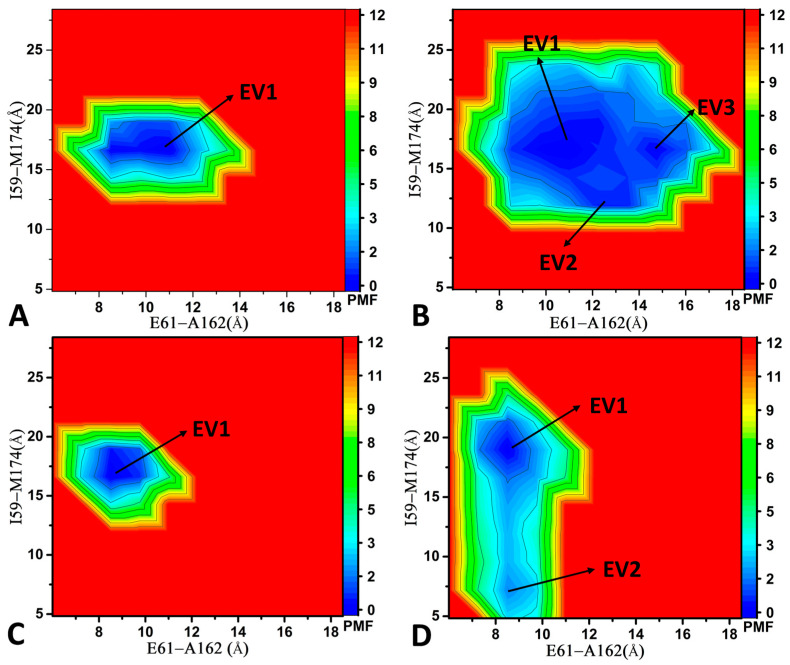
FELs constructed by using the distance of the Cα atom of residue E61 and that of A162 and the distance of the Cα atom of residue I59 and that of M174 as RCs: (**A**) LQQ-bound CDK6/Vcyclin, (**B**) LQQ-bound CDK6, (**C**) AP9-bound CDK6/Vcyclin, and (**D**) AP9-bound CDK6. The PMF is scaled in kcal/mol.

To further explore the impact of Vcyclin protein binding on the conformation of CDK6, we built FELs from another two RCs, namely the distance of the Cα atom of residue E61 in the αC away from that of A162 in the T-loop and the distance of the Cα atom of residue I59 in the αC away from that of M174 in the T-loop. Note that E61, A162, I59, and M174 were obtained from the DL results where their gradient is 0.9. For the LQQ-bound CDK6/Vcyclin and AP9-bound CDK6/Vcyclin, only an energy valley (EV1) was captured by GaMD simulation, and it was situated at (E61-A162, I59-M174) of (10.6 Å, 16.8 Å) and (8.7 Å, 16.9 Å), respectively (Figure 6A,C), implying that the binding of inhibitors does not lead to conformational rearrangement of CDK6 complexed with Vcyclin. For the LQQ-bound CDK6, three energy valleys (EV1-EV3) were captured by GaMD simulations and they were located at the (E61-A162, I59-M174) of (11.0 Å, 17.3 Å), (12.8 Å, 12.4 Å), and (14.7 Å, 16.7 Å) (Figure 6B). For the AP9-bound CDK6, two energy valleys (EV1-EV2) were identified by GaMD simulations (Figure 6D). EV1 and EV2 were, respectively, situated at the (E61-A162, I59-M174) of (8.5 Å, 19.0 Å) and (8.5 Å, 7.2 Å) (Figure 6D). By comparison to the LQQ-bound CDK6/Vcyclin and AP9-bound CDK6/Vcyclin, without binding of the Vcyclin protein, single CDK6 induces more disordered states of the αC helix and the T-loop, verifying that binding of the Vcyclin protein plays an important role in the stabilization of the αC helix and the T-loop conformations, which is supported by the previous study [3,12].

**Figure 7:**
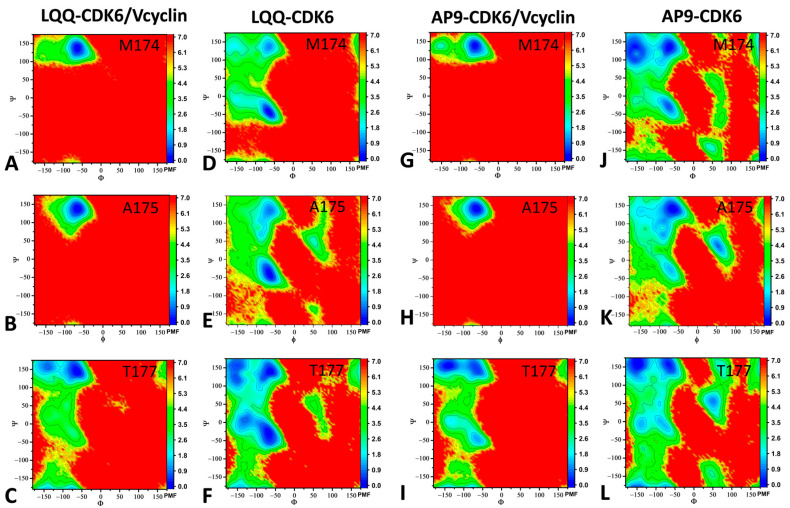
FELs constructed by using the ψ and φ angles of residues M174, A175, and T177 as RCs: (**A**–**C**): FELs of LQQ-bound CDK6/Vcyclin, (**D**–**F**): FELs of LQQ-bound CDK6, (**G**–**I**): FELs of AP9-bound CDK6/Vcyclin, (**J**–**L**): FELs of AP9-bound CDK6. The PMF is scaled in kcal/mol.

The ψ and φ angles of residues M174, A175, and T177 in the T-loop were used to build FELs to study the change conformations of the T-loop, as depicted in Figure 7. Residues M174, A175, and T177 were obtained from the DL results where the gradient of these three residues is 0.9. For the LQQ-bound CDK6/Vcyclin, residue M174 has only one energy valley (EV1), and it is situated at (φ, ψ) of (−62°, 139°) (Figure 7A). Residue A175 also has one energy valley (EV1), and it is situated at (φ, ψ) of (−65°, 137°) (Figure 7B). Residue T177 has two energy valleys (EV1-EV2), and they are situated at (φ, ψ) of (−65°, 145°) and (−140°, 158°), respectively (Figure 7C). With respect to the LQQ-bound CDK6 without Vcyclin, residue M174 has two energy valleys (EV1-EV2), and they are situated at (φ, ψ) of (−66°, −44°) and (−63°, 136°), respectively (Figure 7D). Residue A175 has two energy valleys (EV1–EV2), and they are situated at (φ, ψ) of (−62°, −41°) and (−64°, 133°), respectively (Figure 7E). Residue T177 has five energy valleys (EV1–EV5), and they are situated at (φ, ψ) of (−64°, 142°), (−149°, 157°), (−156°, 122°), (−67°, −35°), and (−130°, 5.9°), respectively (Figure 7F). Compared to the LQQ-bound CDK6/Vcyclin, the dihedral angles of residues M174, A175, and T177 have more states, indicating that the binding of Vcyclin can stabilize the backbone conformations of these three residues.

For the AP9-bound CDK6/Vcyclin, residue M174 has only one energy valley (EV1), and it is situated at (φ, ψ) of (−61°, 137°) (Figure 7G). Residue A175 also has one energy valley (EV1), and it is situated at (φ, ψ) of (−65°, 138°) (Figure 7H). Residue T177 has three energy valleys (EV1-EV3), and they are situated at (φ, ψ) of (−64°, 147°), (−144°, 156°), and (−65°, −44°), respectively (Figure 7I). Compared to the LQQ-bound CDK6/Vcyclin, the AP9-bound CDK6/Vcyclin has similar FELs for residue M174 and residue A175, with an additional EV at residue T177. With regard to the AP9-bound CDK6, residue M174 has three energy valleys (EV1-EV3), and they are situated at (φ, ψ) of (−154°, 135°), (−66°, 136°), and (−66°, −28°), respectively (Figure 7J). Residue A175 has four energy valleys (EV1-EV4), and they are situated at (φ, ψ) of (−65°, 139°), (−89°, 70°), (53°, 36°), and (−72°, −25°), respectively (Figure 7K). Residue T177 has five energy valleys (EV1-EV5), and they are situated at (φ, ψ) of (−62°, 158°), (−150°, 157°), (−88°, −0.3°), (−138°, −7.6°), and (52°, 58°), respectively (Figure 7L). Compared to the AP9-bound CDK6/Vcyclin, the dihedral angles of residues M174, A175, and T177 show more conformational states, implying that the binding of Vcyclin can stabilize the backbone conformations of these three residues.

Based on the aforementioned results, the conformation of the binding pocket and the interface of the Vcyclin protein are significantly affected by the binding of inhibitors and the Vcyclin proteins: (1) the up–down moving of the G-loop results in the open and closed states of the binding pocket; (2) unlike the binding pocket of the LQQ-bound CDK6, that of AP9-bound CDK6 has no closed state of the binding pocket and (3) without the Vcyclin protein, single CDK6 leads to more disordered states of the αC helix and the T-loop. The work of He et al. showed that the flexibility of the G-loop alters the conformation of the binding pocket, supporting our current findings [71]. The study of Schulze-Gahmen et al. indicated that the Vcyclin protein contributes to the stable conformation of the T-loop [3], agreeing with our current results.

### 2.3. Dynamics Behavior of CDK6

**Figure 8:**
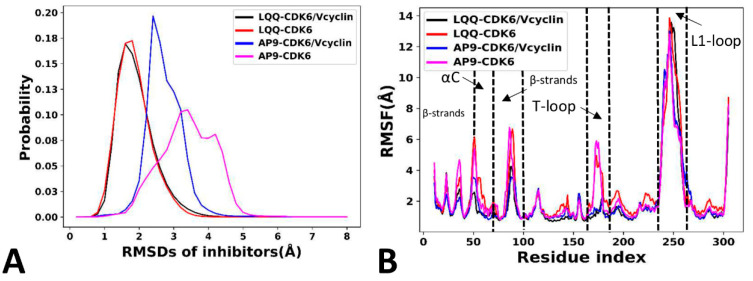
Dynamics indexes: (**A**) the probability distribution of inhibitors’ RMSDs, (**B**) RMSFs of CDK6.

To understand the different structural stabilities of inhibitors, root-mean-square deviations (RMSDs) of heavy atoms for LQQ and AP9 were calculated relative to the initial structure (Figure 8A and Figure S2). It is observed that the inhibitors show a stable fluctuation in the LQQ-bound CDK6/Vcyclin, the LQQ-bound CDK6, and the AP9-bound CDK6/Vcyclin systems. The RMSDs of LQQ in the LQQ-bound CDK6/Vcyclin and the LQQ-bound CDK6 systems are populated at 1.6 Å and 1.9 Å (Figure 8A), respectively. The RMSD of AP9 in the AP9-bound CDK6/Vcyclin system is populated at 2.4 Å (Figure 8A), while the RMSD of AP9 in the AP9-bound CDK6 is populated at two peaks of 3.4 and 4.2 Å (Figure 8A), with a wider distribution range, suggesting that the absence of the Vcyclin protein increases the RMSD of AP9. Meanwhile, we also calculate the RMSDs of all heavy atoms from proteins to understand the structural stability of proteins (Figure S3). The results show that proteins show a similar fluctuation and structural stability through GaMD simulations. Root-mean-square fluctuations (RMSFs) of CDK6 were estimated by using the coordinates of the Cα atoms (Figure 8B). The LQQ-bound CDK6 and AP9-bound CDK6 strengthen the structural flexibility of most of the CDK6 regions, particularly in the β-strands, the αC helix, and the T-loop relative to the LQQ-bound CDK6/Vcyclin and AP9-bound CDK6/Vcyclin. It is also found that the L1-loop shows stronger flexibility (Figure 8B). Furthermore, it is worth noting that the residues obtained from the DL results are also located within these regions. These results indicate that the changes in the structural flexibility can influence the function of CDK6.

**Figure 9:**
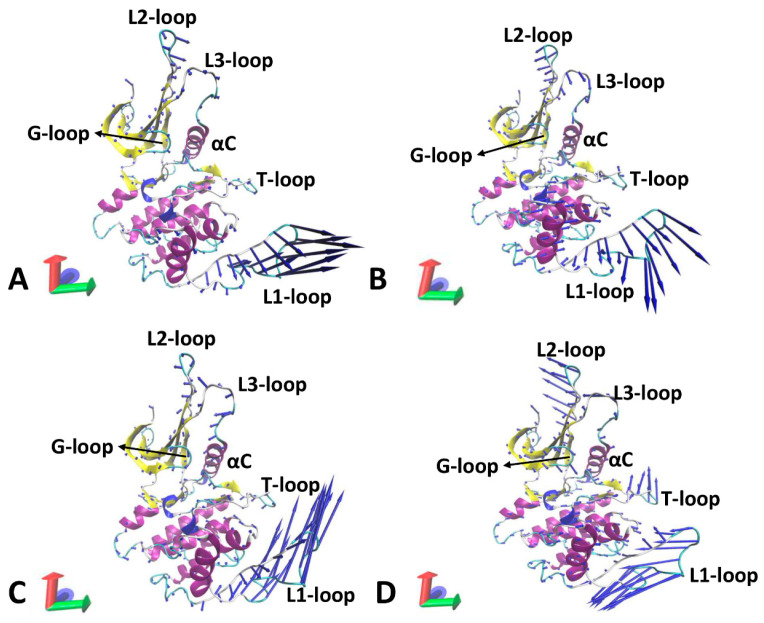
Concerted motions of structural domains from CDK6 revealed by PCA: (**A**) the LQQ-bound CDK6/Vcyclin, (**B**) the LQQ-bound CDK6, (**C**) the AP9-bound CDK6/Vcyclin, and (**D**) the AP9-bound CDK6. CDK6 was shown in cartoon modes.

To explore the impacts of the inhibitor and Vcyclin binding on the concerted movements of structural domains in CDK6, PCA was performed on the coordinates of the Cα atoms using the CPPTRAJ module in Amber 20. The function of eigenvalues over eigenvectors is depicted in Figure S4, which is used to characterize the structural fluctuation along the eigenvectors. It is found that the binding of the Vcyclin protein weakens the total structural fluctuation of CDK6. The first eigenvector was visualized utilizing the VMD software 1.9.4a51 [72] and the results are displayed in Figure 9. Structural domains of CDK6 display well-concerted motions; furthermore, the Vcyclin protein greatly affects the concerted motions of the L1-loop. In the LQQ-bound CDK6/Vcyclin, the L2-loop and L3-loop generate a parallel concerted motion in the same direction. It was also found that the L1-loop exhibits a high fluctuation (Figure 9A). Differently, in the LQQ-bound CDK6, the L2-loop and L3-loop have an opposite fluctuation tendency and the L1-loop has a different motion direction (Figure 9B). By referencing the LQQ-bound CDK6/Vcyclin, removal of the Vcyclin protein slightly strengthens the fluctuations of the α helices in the C-lobe domains. Compared to the AP9-bound CDK6/Vcyclin (Figure 9C), the removal of Vcyclin highly strengthens the concerted motions of the L2-loop and T-loop (Figure 9C). By comparison with the AP9-bound CDK6/Vcyclin, cutting the Vcyclin changes the fluctuation tendency of the L1-loop (Figure 9D). In summary, the removal of the Vcyclin protein greatly alters the structural fluctuation of the T-loop, L1-loop, and L2-loop, implying that these structural domains are possibly involved in the function domains of CDK6.

### 2.4. Binding Free Energy Calculations

To explore the binding preference of two inhibitors, the binding free energies of LQQ and AP9 to CDK6 with and without the binding of Vcyclin were calculated with the QM/MM-GBSA method. The calculated free energy components are listed in Table 1. It is worth noting that the rank of the experimental values is consistent with that of the binding free energies predicted by the QM/MM-GBSA [66], which suggests that our current analyses on free energy are reliable.

The inhibitor–CDK6 van der Waals interactions (${∆E}_{vdW}$) in the LQQ-bound CDK6/Vcyclin, LQQ-bound CDK6, AP9-bound CDK6/Vcyclin, and AP9-bound CDK6 are −52.14, −51.73, −48.89, and −42.90 kcal/mol, respectively. On the whole, the binding ability of LQQ to CDK6 is stronger than that of AP9. By comparison with CDK6 bounded by Vcyclin, it is found that the removing of the Vcyclin protein slightly weakens the van der Waals interactions of LQQ and AP9 with CDK6. The electrostatic interactions (${∆E}_{ele}$) provide few contributions for inhibitor binding. The self-consistent field energy (${∆G}_{scf}$) of AP9-bound CDK6/Vcyclin and AP9-bound CDK6 are abated by 5.1 and 7.76 kcal/mol relative to those of LQQ-bound CDK6/Vcyclin and LQQ-bound CDK6, which shows that HBI between V101 and the inhibitor plays a key role in the binding of the inhibitor to CDK6. The binding entropies (−T∆S) of LQQ-bound CDK6/Vcyclin, LQQ-bound CDK6, AP9-bound CDK6/Vcyclin, and AP9-bound CDK6 are 17.20, 20.98, 21.08, and 19.22 kcal/mol, respectively, which are unfavorable for the binding of inhibitors to CDK6. On the whole, the binding abilities of inhibitors LQQ and AP9 to CDK6 are strengthened by 1.62 and 2.24 kcal/mol because of the Vcyclin binding. The results not only suggest that the binding ability of LQQ to CDK6 is stronger than AP9 to CDK6 but also verifies that the binding of the Vcyclin protein strengthens the binding ability of the inhibitor to CDK6. In summary, van der Waals interactions and HBI contribute the main force to inhibitor–CDK6 binding. In the future drug design with regard to CDK6, these two interactions should be paid special attention.

### 2.5. Analyses of Inhibitor–CDK6 Interaction Networks

**Figure 10:**
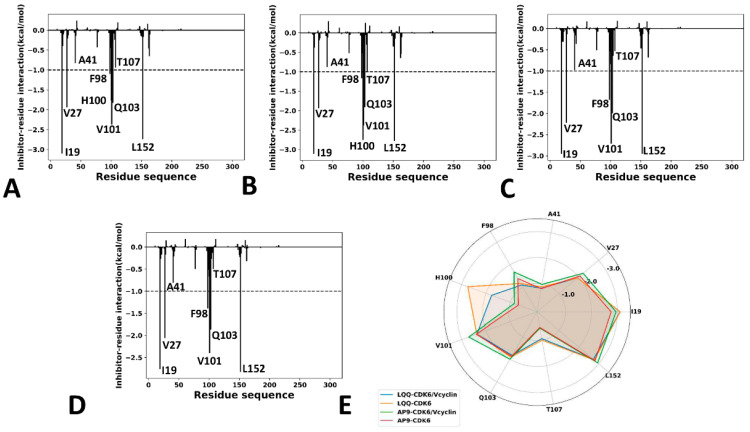
Interaction of inhibitors with CDK6: (**A**) interaction spectrum of the LQQ-bound CDK6/Vcyclin, (**B**) interaction spectrum of the LQQ-bound CDK6, (**C**) interaction spectrum of the AP9-bound CDK6/Vcyclin, (**D**) interaction spectrum of the AP9-bound CDK6, and (**E**) key residues in inhibitor–CDK6 interactions.

**Figure 11:**
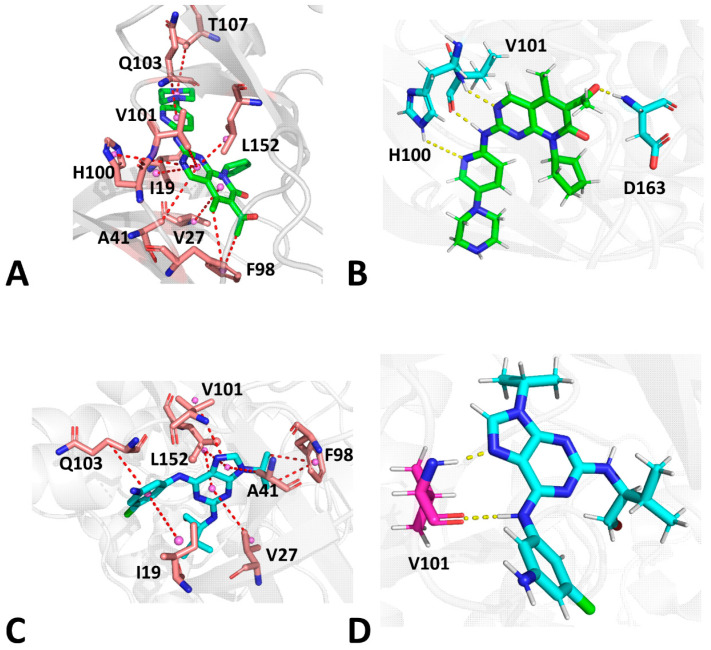
Geometric information of inhibitor–residue interactions: (**A**) the hydrophobic interactions of LQQ with CDK6, (**B**) the LQQ-CDK6 HBIs, (**C**) the hydrophobic interactions between AP9 and residues, and (**D**) the AP9-CDK6 HBIs.

To obtain atomic-level insights into the interaction modes of inhibitors with CDK6, the residue-based free energy decomposition method was applied to estimate the inhibitor–residue interaction spectrum of LQQ and AP9 with CDK6, and the results are displayed in Figure 10. The contributions of the side chains and backbones of residues to the inhibitor–CDK6 associations are provided in Table 2. HBIs between the inhibitors and residues of CDK6 were analyzed using the program CPPTRAJ, and the results are listed in Table 3. The geometric information regarding inhibitor–residue interactions is depicted in Figure 11.

For the LQQ-bound CDK6/Vcyclin, LQQ produces interactions stronger than −0.8 kcal/mol with nine residues, including I19, V27, A41, F98, H100, V101, Q103, T107, and L152 (Figure 10A,E). According to Figure 11A, the hydrophobic groups of I19, V27, A41, H100, V101, Q103, T107, and L152 are located near the hydrophobic ring of LQQ. Thus, H100 structurally forms the π-π interaction of −1.77 kcal/mol with LQQ. Residues I19, V27, A41, V101, Q103, T107, and L152 yield the CH-π interactions with LQQ, and their corresponding interaction energies are −3.1, −1.94, −0.83, −2.37, −1.83, −0.94, and −2.74 kcal/mol, respectively (Figure 10A,E and Table 2). Structurally, the phenyl groups of F98 are adjacent to the alkyls of LQQ, which leads to a CH-π interaction of −1.1 kcal/mol (Figure 11A and Table 2). In addition, H100 and V101 form three HBIs with LQQ, and their occupancy is higher than 65.4% (Table 3), indicating that these three hydrogen bonds are stable though the entire GaMD simulations. According to Table 2, the energetic contributions of I19, V27, A41, F98, T107, and L152 mostly arise from the sidechains of these residues. The energetic contribution of H100 mainly comes from the van der Waals interactions of its sidechain but the polar interactions are weak. The energetic contribution of V101 mostly arises from the van der Waals interactions of its sidechain and electrostatic interaction of its backbone. The energetic contribution of Q103 is primarily provided by the van der Waals interactions of both the sidechain and the backbone of Q103. Moreover, a hydrogen bond with an occupancy of 53% appears between LQQ and D163 (Table 3), implying a favorable force for the LQQ-CDK6 binding. Compared to the LQQ-bound CDK6/Vcyclin, LQQ produces similar interaction modes with CDK6 in the LQQ-bound CDK6, but the deleting of the Vcyclin protein also induces the alteration. By referencing the LQQ-bound CDK6/Vcyclin, the removal of the Vcyclin protein strengthens the LQQ-H100 interaction (Table 2), which mainly comes from the changes in electrostatic interactions of the sidechain from H100 with LQQ.

For the AP9-bound CDK6/Vcyclin, AP9 produces interactions stronger than −0.8 kcal/mol with seven residues, including I19, V27, A41, F98, V101, Q103, and L152 (Figure 10C,E). According to Figure 11C, the alkyls of I19, V27, A41, V101, and L152, and the CH-group of Q103 are located near the hydrophobic ring of AP9. Thus I19, V27, A41, V101, Q103, and L152 yield the hydrophobic CH-π interactions, and their corresponding interaction energies are −2.95, −2.22, −0.98, −2.71, −2.0, and −2.95 kcal/mol, respectively (Figure 10C,E and Table 2). Structurally, the phenyl groups of F98 are adjacent to the alkyls of AP9, which leads to a CH-π interaction of −1.68 kcal/mol (Figure 11C and Table 2). According to Table 2, the energetic contributions of I19, V27, A41, F98, and L152 mostly arise from the sidechains of these residues. The energetic contribution of V101 mainly stems from the van der Waals interactions of its sidechain with AP9 and the electrostatic interaction of its backbone with AP9. The energetic contribution of Q103 arises from the van der Waals interactions of both the sidechain and the backbone of Q103 with AP9. In addition, AP9 forms two HBIs with V101, and their occupancy is higher than 59.7%, indicating that these two hydrogen bonds are stable through the entire GaMD simulations (Table 3). By comparison with the AP9-bound CDK6/Vcyclin, AP9 yields the same interaction modes in the AP9-bound CDK6. Apart from residue Q103, the removal of the Vcyclin protein slightly weakens the interactions of AP9 with I19, V27, A41, F98, V101, and L152 (Table 2). The deletion of the Vcyclin protein enhances the occupancy of the hydrogen bond V101-N-H…AP9-N7 relative to the AP9-bound CDK6/Vcyclin, but decreases the occupancy of the hydrogen bond V101-O…AP9-N6-H6.

Based on the aforementioned description, two inhibitors, LQQ and AP9, form hydrophobic interactions with common residues I19, V27, A41, F98, V101, Q103, T107, and L152 of CDK6; moreover, our current predicted binding sites are in good agreement with the experiment results [66]. Differently, LQQ also forms hydrophobic interaction with residue H100. By comparison with LQQ, AP9 misses two HBIs with residues H100 and D163. These different interactions reflect the structural difference between LQQ and AP9. It is concluded that the above-mentioned residues play key roles in the binding of inhibitors to CDK6. More importantly, the CH-π and π-π interactions, and HBIs between the above-mentioned residues and inhibitors are identified as target sites of drug design with regard to CDK6, which should be paid special attention.

## 3. Materials and Methods

### 3.1. Scheme of Operating Calculations

**Figure 12:**
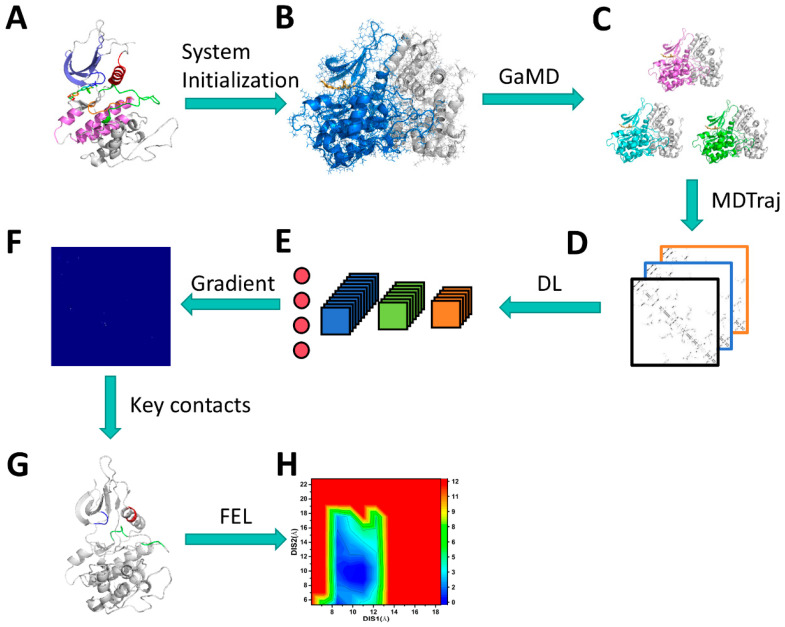
Workflow of deep learning from GaMD simulations: (**A**) the structure of CDK6, (**B**) the initialized system bound with the inhibitor, (**C**) conformational ensembles recorded in three independent GaMD trajectories, (**D**) images extracted from the MDTraj program, (**E**) convolution neural networks, (**F**) the saliency maps obtained from backward propagation, (**G**) key residue contacts identified by deep learning, and (**H**) free energy landscapes used for revealing the conformation of CDK6 influenced by the inhibitors and the Vcyclin protein.

The integration of MS-GaMD simulations and DL was employed to identify crucial residue contacts and uncover significant function domains of CDK6 binding. The overall approach is illustrated in Figure 12. The procedure involved: (1) extracting initial atomic coordinates from the PDB and constructing simulation systems using the Amber program with force field parameters, (2) performing three separate GaMD simulations to relax conformations and gather conformational ensembles, (3) utilizing the MDTraj program to convert conformational ensembles into images suitable for DL analysis, (4) randomly dividing the images into training and validation sets for image classification based on a two-dimensional (2D) convolutional neural network (CNN), (5) identifying significant residue contacts through the gradient maps, and finally, (6) obtaining reaction coordinates (RCs) from these key residue contacts to construct FELs and reveal conformation changes of CDK6.

### 3.2. Constructions of Simulated Systems

The initial structures of the LQQ-bound CDK6/Vcyclin and AP9-bound CDK6/Vcyclin complexes were obtained from the PDB and they, respectively, correspond to the PDB entries 2EUF and 2F2C [66]. The LQQ-bound CDK6 without the Vcyclin protein was obtained by cutting Vcyclin from the crystal structure 2EUF. The AP9-bound CDK6 without the Vcyclin protein was obtained by deleting Vcyclin from the crystal structure 2F2C. The missing residues of CDK6 in four crystal structures were repaired by using the program Modeller [73]. All of the crystal water and non-inhibitor molecules were deleted from the initial model. The protonated states of residues in CDK6 were examined by using the program H++3.0 [74]. The Leap module in Amber20 [75,76] was utilized to complete the following tasks: (1) assigning force field parameters for CDK6 and CDK6/Vcyclin using the *ff*19SB force field, (2) constructing a periodic box of octahedral TIP3P water molecules with a buffer size of 10.0 Å to solve four CDK6-related systems, and (3) adding counter ions into each system within a 0.15 M salt environment to achieve neutralization, where the force parameters for sodium ions (Na^+^) and chloride ions (Cl^−^) were obtained from Joung and Cheatham’s work [77,78]. The molecular structures of two inhibitors, LQQ and AP9, were optimized at a semi-empirical AM1 level, followed by assigning BCC charges to each atom of the inhibitors using Amber20’s Antechamber module [79]. The general Amber force field (GAFF2) [80,81] was employed to generate force field parameters for both LQQ and AP9 inhibitors.

### 3.3. Multiple Separate Gaussian Accelerated Molecular Dynamics

With the expectation of relieving bad contacts between atoms caused by the initialization of four current CDK6-related systems, each system endures two-step minimizations consisting of 50,000-step steepest descent minimization and 50,000-cycle conjugate gradient minimization. The optimized systems were slowly heated from 0 to 300 K within 1 ns in the canonical ensemble (NVT) with a weak harmonic restriction of 2 kcal mol^−1^·Å^2^ on non-hydrogen atoms and inhibitors. Then, the four systems were further equilibrated at 300 K within 1 ns under the isothermal−isobaric ensemble (NPT). Subsequently, the 2 ns NPT conduction was carried out to keep the density of the system at approximately 1.01 g/cm^3^. Finally, three separate 6.6 ns cMD productions were performed on each system at the NVT with periodic boundary conditions and the particle mesh Ewald method (PME) [82], during which the initial atomic velocities of each structure were assigned by means of the Maxwell distribution. Three well-equilibrated ending structures of each system were used as starting points for running three separate GaMD simulations on each system.

GaMD enhances conformational sampling of biomolecules by increasing the harmonic enhancement potential, thereby smoothing the potential energy surface of the system. A system with *N* atoms is located in $\overset{⃑}{r}=\{{\overset{⃑}{r}}_{1},\ldots ,{\overset{⃑}{r}}_{n}\}$, and when the system potential is lower than a reference energy *E*, it is raised by:

(1) $$∆V\left(\overset{⃑}{r}\right)=\left\{\begin{array}{cc} 0, & V(\overset{⃑}{r})\geq E\\ \frac{1}{2}k{\left(E-V\left(\overset{⃑}{r}\right)\right)}^{2}, & V\left(\overset{⃑}{r}\right)<E \end{array}\right.$$

where *k* represents the harmonic force constant. The biased system potential is:

(2) $$V^{*}\left(\overset{⃑}{r}\right)=\left\{\begin{array}{cc} V(\overset{⃑}{r}), & V(\overset{⃑}{r})\geq E\\ V\left(\overset{⃑}{r}\right)+\frac{1}{2}k{\left(E-V\left(\overset{⃑}{r}\right)\right)}^{2}, & V\left(\overset{⃑}{r}\right)<E \end{array}\right.$$

Three conditions should be satisfied to smooth the potential energy surface of enhanced sampling. First, for any two potential energies, $V_{1}\left(\overset{⃑}{r}\right)$ and $V_{2}\left(\overset{⃑}{r}\right),$ from the original energy surface, if $V_{1}\left(\overset{⃑}{r}\right)\;{<V}_{2}\left(\overset{⃑}{r}\right)$, ∆V should be a monotonic function that means $V_{1}^{*}\left(\overset{⃑}{r}\right)<V_{2}^{*}\left(\overset{⃑}{r}\right)$. Substituting $V_{1}^{*}\left(\overset{⃑}{r}\right)<V_{2}^{*}\left(\overset{⃑}{r}\right)$ into Equation (2), we can yield:

(3) $$E<\frac{1}{2}\left[V_{1}\left(\overset{⃑}{r}\right)+V_{2}\left(\overset{⃑}{r}\right)\;\right]+\frac{1}{k}$$

Second, if $V_{1}\left(\overset{⃑}{r}\right)\;{<V}_{2}\left(\overset{⃑}{r}\right)$, the potential difference observed on the smoothed energy surface should be smaller than that of the original, meaning $V_{2}^{*}\left(\overset{⃑}{r}\right)-V_{1}^{*}\left(\overset{⃑}{r}\right)<V_{2}\left(\overset{⃑}{r}\right)-V_{1}\left(\overset{⃑}{r}\right)$. Similarly, substituting $V_{2}^{*}\left(\overset{⃑}{r}\right)-V_{1}^{*}\left(\overset{⃑}{r}\right)<V_{2}\left(\overset{⃑}{r}\right)-V_{1}\left(\overset{⃑}{r}\right)$ into Equation (2), we can generate:

(4) $$E>\frac{1}{2}\left[V_{1}\left(\overset{⃑}{r}\right)+V_{2}\left(\overset{⃑}{r}\right)\;\right]$$

Combining Equations (3) and (4), the reference energy *E* should be satisfied in the following range:

(5) $$V_{max}\leq E\leq V_{min}+\frac{1}{k}$$

in which $V_{max}$ and $V_{min}$ are the system maximum and minimum potential energies. To ensure Equation (5), *k* must satisfy:

(6) $$k\leq \frac{1}{V_{max-}V_{min}}$$

Then, we can define k as

(7) $$k=k_{0}\cdot \left(\frac{1}{V_{max-}V_{min}}\right)\;,\;0\;\leq k\leq 1$$

Third, the standard deviation of ∆V should be sufficiently small to ensure the appropriateness of energetic reweighting:

(8) $$\sigma_{∆V}^{2}=k\cdot \left(E-V_{avg}\right),\;\sigma_{V}<\sigma_{0}$$

where $\sigma_{∆V}$ is the standard deviation of ∆V, $V_{avg}$ is the average potential energy, and $\sigma_{V}$ is the standard deviation of the potential energy. $\sigma_{0}$ is a user-defined upper limit for appropriate reweighting. When *E* is set to the lower bound $E=V_{max}$, $k_{0}$ can be:

(9) $$k_{0}=min(1.0,\frac{V_{max}-V_{min}}{V_{max}-V_{avg}})$$

Similarly, when *E* is set to the upper bound $E=V_{min}+\frac{1}{k}$, $k_{0}$ can be:

(10) $$k_{0}\geq (1.0-\frac{\sigma_{0}}{\sigma_{V}})\cdot (\frac{V_{max}-V_{min}}{V_{avg}-V_{min}})$$

in which $k_{0}$ is the effective harmonic constant that determines the magnitude of the applied push potential. As $k_{0}$ increased, a higher harmonic push potential was applied to the free energy surface, thus enhancing the conformational sampling of biomolecules. Before GaMD simulation, a cMD simulation is conducted to obtain the $V_{max}$, $V_{min}$, $V_{avg}$, and $\sigma_{V}$ of each simulation system. In our current study, 3 µs GaMD simulations were run on the LQQ-bound CDK6/Vcyclin, LQQ-bound CDK6, AP9-bound CDK6/Vcyclin, and AP9-bound CDK6 systems. These simulations consisted of three separate GaMD simulations, each running for 1 µs. To facilitate data analysis, we combined three separate GaMD trajectories into a single GaMD trajectory (SGT) using the CPPTRAJ module in Amber 20 [83]. This allows us to extract valuable insights into the function of CDK6. We employed a program called PyReweighting developed by Miao et al. [84] to accurately reweight and identify the original free energy profiles of our CDK6-related systems. The SHAKE algorithm was used in both cMD and GaMD simulations to constrain chemical bonds between hydrogen atoms and heavy atoms [85]. To regulate the temperatures of four CDK6-related systems, we utilized a Langevin thermostat with a collision frequency of 1.0 ps^−1^ [86]. Non-bonded interactions were estimated using the PME method with a cutoff distance of 9 Å. All simulations were performed using pmemd.cuda implemented in Amber 20 [75,76].

### 3.4. Deep Learning

Deep Learning was employed to analyze the GaMD simulations of the four CDK6-related system. Contact maps for the conformational frames of the GaMD simulations were computed using a contact definition of ≤4.5 Å between any Cα atoms in a time interval of 100 ps. The Python packages MDTraj [87] and contact map explorer [87] were utilized to generate 295 × 295 residue contact maps. These contact maps were transformed into grayscale images of 295 × 295 pixels for subsequent 2D CNN analysis. A total of 30,000 images were generated for each CDK6-related system, with a random selection of 80% images used for training and the remaining images used for validation purposes. Our constructed 2D CNN was implemented using the Python PyTorch 1.12.1 package. The optimal model consisted of four convolutional layers with kernel sizes of 3 × 3, comprising filters in quantities of 32, 32, 64, and 64, respectively, followed by two fully connected (dense) layers where the first two contained filters numbering at 512 and then 128, respectively; both layers had a dropout rate set at 0.5. The final fully connected layer was the classification layer, which categorized input data into four classes: LQQ-bound CDK6/Vcyclin, LQQ-bound CDK6, AP9-bound CDK6/Vcyclin, and AP9-bound CDK6. Throughout all layers of the 2D CNN, except for the classification layer, the “ReLU” activation function was employed, whereas in the classification layer, the “softmax” activation function was utilized. The “softmax” function transforms the network’s output into a probability distribution that represents the likelihood of each potential class. A 2 × 2 max-pooling layer was added after each convolutional layer. Finally, by utilizing the residue contact maps from the most populated structure in each CDK6-related system, significance (attention) maps of residue contact gradients were computed through backpropagation. We declare that our program was rewritten with the PyTorch package based on the work of Miao’s group [63,64].

### 3.5. Construction of Free Energy Landscapes

Key residue contacts were detected by the DL model according to the three following criteria: (1) the contact gradients were higher than 0.7 in the saliency maps, (2) the residue contacts were calculated by using all Cα atoms, and (3) obvious changes in contacts among structural domains can be captured. The distances between the Cα atoms of key residues were used as RCs to construct FELs according to the reweighting procedure.

In the reweighting process of GaMD simulations, the reweighted free energy $F\left(A\right)=-k_{B}Tln(\rho_{A})$ can be calculated as:

(11) $$F\left(A\right)=F^{*}\left(A\right)-\sum_{k=1}^{2}\frac{\beta^{k}}{k!}C_{k}+F_{C}$$

where $F^{*}\left(A\right)=-k_{B}Tlnp^{*}\left(A\right)$ represents the modified free energy arising from GaMD simulations, $F_{C}$ denotes a constant, and ${\beta =k}_{B}T.$ The probability distribution $p^{*}\left(A\right)$ of selected RCs from GaMD simulations can be reweighted to recover the canonical ensemble distribution $\rho_{A}$. All calculations involved in the free energy reweighting were realized by using the program PyReweighting 1.0 developed by Miao et al., and the details for the reweighting procedure have been elucidated in the work of Miao et al. [84].

### 3.6. Principal Component Analysis

PCA is a valuable tool for gaining insights into concerted motions of structural domains within biomolecules. Hence, we employed PCA to clarify how the binding of inhibitors and Vcyclin protein impacts the concerted motions of CDK6. In our work, PCA was performed by diagonalizing a covariance matrix C, which was constructed using the coordinates of the Cα atoms in CDK6 based on Equation (12):

(12) $$C=\langle (q_{i}-\langle q_{i}\rangle )({q_{j}-\langle q_{j}\rangle }^{T})\rangle$$

in which $q_{i}$ and $q_{j}$ represent the Cartesian coordinates of the *i*th and *j*th Cα atoms in CDK6, respectively, while $\langle q_{i}\rangle$ and $\langle q_{j}\rangle$ are their averaged positions obtained from conformational ensembles recorded at the SGT. To calculate this average, a superimposition of the SGT with a referenced structure is performed to eliminate overall translations and rotations using a least-squares fitting procedure [88]. The resulting eigenvalues and eigenvectors from the PCA are usually applied to, respectively, embody the fluctuation amplitude along an eigenvector and the concerted motions of structural domains. In our study, we performed the PCA using the CPPTRAJ program in Amber 20 [83].

### 3.7. QM/MM-GBSA Calculations

Binding free energies are usually employed to evaluate the binding ability of inhibitors to their targets. Inhibitor binding is influenced by two crucial factors, namely enthalpy changes and entropy changes. Currently, molecular mechanics Poisson–Boltzmann surface area (MM-PBSA) and MM-GBSA are considered as two effective methods for this purpose [80,89]. Hou’s team performed several works to evaluate the performance of these two methods [90,91,92]. Chen’s group proved that the QM/MM-GBSA method can accurately evaluate the hydrogen bonding interactions [93]. Based on their information, we selected the QM/MM-GBSA method to calculate inhibitor–CDK6 binding free energies with Equation (13) as follows:

(13) $${∆G}_{bind}=∆E_{ele}+∆E_{vdw}+∆G_{gb}+∆G_{surf}+∆G_{scf}-T∆S$$

where $∆E_{ele}$ and $∆E_{vdw}$ indicate the electrostatic and van der Waals interactions between the inhibitor and CDK6, which were obtained from the Amber force field *ff19SB*. The term $∆G_{surf}$ was estimated by utilizing the empirical equation $∆G_{surf}=\gamma \times ∆SASA+\beta$ that involves the solvent-accessible surface area ∆SASA, with γ and β setting as 0.0072 kcal·mol·Å^−2^ and 0.0 kcal·mol^−1^, respectively [69,94]. $∆G_{gb}$ was estimated using the generalized Born (GB) model [95]. The $∆G_{scf}$ indicates the self-consistent field energy. The V101 residue involving a hydrogen binding interaction (HBI) is described at the QM level using the semi-empirical Hamiltonian PM3 method to treat this HBI. All of the other systems were characterized at the molecular mechanics level. The contribution of the entropic changes, T∆S, was computed through the mmpbsa_py_nabnmode program in Amber 20 [96]. To calculate binding enthalpy, we extracted 400 snapshots from cMD simulations. Due to computational expense, 50 snapshots from the above-mentioned 400 snapshots were utilized for entropy calculations.

## 4. Conclusions

CDK6 plays a key role in the regulation of the cell cycle and is considered a crucial target for cancer therapy. Three separate GaMD simulations, each running for 1 us, were conducted on each system to probe the conformational dynamics of inhibitor-bound CDK6. CNN-based DL was performed to identify significant function domains, and the results reveal that the binding pocket and T-loop binding to the Vcyclin protein are involved in obvious differences in structural contacts. The RCs learned by DL were used to construct FELs, and the results indicate that the binding pocket and T-loop lead to more disordered states. The information from PCA suggests that the Vcyclin protein alters the structural fluctuation of the T-loop. The interaction networks between the inhibitor and CDK6 residues uncover that the LQQ inhibitor not only has more binding residues but also contributes more HBIs with CDK6. The information on the structural difference between LQQ and AP9 is useful for the structural optimization of inhibitor molecules. The results from the QM/MM-GBSA calculations not only suggest that the binding ability of LQQ to CDK6 is stronger than AP9 to CDK6 but also verifies that the binding of the Vcyclin protein strengthens the binding ability of an inhibitor to CDK6. This work is also anticipated to provide useful theoretical aids for the understanding of the function of CDK6.

## Figures and Tables

**Table 1:** Binding free energies of inhibitors to CDK6 estimated by utilizing the QM/MM-GBSA method.

^a^ Components	LQQ-Bound CDK6/Vcyclin		LQQ-Bound CDK6		AP9-Bound CDK6/Vcyclin		AP9-Bound CDK6	
	Average	^b^ STD	Average	STD	Average	STD	Average	STD
^c^ ${∆E}_{ele}$	0.09	0.00	0.07	0.00	0.08	0.00	0.08	0.00
^c^ ${∆E}_{vdW}$	−52.14	0.21	−51.73	0.21	−48.89	0.17	−42.90	0.22
^c^ ${∆G}_{gb}$	30.43	0.32	28.75	0.32	29.68	0.44	25.33	0.43
^c^ ${∆G}_{surf}$	−5.90	0.02	−5.95	0.02	−5.90	0.02	−5.26	0.02
^d^ ${∆G}_{scf}$	−10.72	0.30	−11.54	0.28	−5.62	0.44	−3.78	0.50
^e^ ∆H	−38.23	0.26	−40.39	0.28	−30.62	0.19	−26.52	0.29
^f^ −T∆S	17.20	1.18	20.98	1.05	21.08	1.17	19.22	0.97
^g^ ${∆G}_{bind}$	−21.03		−19.41		−9.54		−7.3	
^h^ ${∆G}_{exp}$	−11.11				−9.09			

^a^ All components of free energies are in kcal/mol. ^b^ Standard errors. ^c^ ${∆E}_{ele}$, ${∆E}_{vdW},\;{∆G}_{gb}$, and ${∆G}_{surf}$ indicate van der Waals interaction, electrostatic interaction, polar solvation, and nonpolar solvation free energies, respectively. ^d^${\;∆G}_{scf}$ indicates the self-consistent field energy in QM region. ^e^ Binding enthalpy: $∆H=∆E_{ele}+∆E_{vdW}+{∆G}_{gb}+{∆G}_{surf\;}+{∆G}_{scf}.\;$^f^ Entropy contribution: −*T*∆S. ^g^ Binding free energy: $∆G_{bind}=∆H-T∆S.$ ^h^ ${∆G}_{exp}$ is obtained via ∆G=−RTlnIC50 with the experimental value of IC50 [66].

**Table 2:** Contributions of the side chains and backbones to inhibitor–residue interaction ^a^.

Compound	Residue	$S_{vdW}$	$B_{vdW}$	$T_{vdW}$	$S_{ele}$	$B_{ele}$	$T_{ele}$	$S_{gb}$	$B_{gb}$	$T_{gb}$	∆G
LQQ-CDK6/Vcyclin	I19	−2.75	−0.95	−3.7	−0.22	−0.32	−0.54	0.18	1.5	1.68	−3.1
	V27	−1.53	−0.15	−1.68	−0.12	−0.07	−0.19	0.11	0.04	0.15	−1.94
	A41	−0.69	−0.2	−0.89	0.02	0.01	0.03	−0.02	0.11	0.09	−0.83
	F98	−1.15	−0.13	−1.28	0.21	0.17	0.38	−0.05	−0.04	−0.09	−1.1
	H100	−1.15	−0.34	−1.49	−0.86	−0.63	−1.49	1.12	0.14	1.26	−1.77
	V101	−0.92	−0.2	−1.12	0.01	−2.17	−2.16	−0.03	1.0	0.97	−2.37
	Q103	−1.02	−0.84	−1.86	−0.17	0.13	−0.04	0.23	−0.02	0.21	−1.83
	T107	−0.68	−0.08	−0.76	−0.53	0.1	−0.43	0.66	−0.22	0.43	−0.94
	L152	−2.32	−0.1	−2.41	−0.04	−0.03	−0.08	0.1	−0.08	0.02	−2.74
LQQ-CDK6	I19	−2.69	−0.62	−3.3	−0.19	−0.23	−0.42	0.2	0.88	1.09	−3.11
	V27	−1.57	−0.15	−1.72	−0.08	−0.02	−0.1	0.09	0.02	0.11	−1.94
	A41	−0.72	−0.22	−0.94	0.01	−0.03	−0.01	−0.01	0.15	0.14	−0.88
	F98	−1.19	−0.13	−1.32	0.22	0.16	0.38	−0.06	−0.06	−0.12	−1.17
	H100	−1.22	−0.34	−1.56	−2.49	−0.72	−3.21	1.88	0.21	2.09	−2.75
	V101	−0.82	−0.18	−1.0	0.0	−2.41	−2.41	−0.02	1.1	1.08	−2.38
	Q103	−0.96	−0.81	−1.77	−0.14	0.05	−0.09	0.16	−0.1	0.07	−1.91
	T107	−0.71	−0.08	−0.79	−0.79	0.16	−0.63	0.92	−0.3	0.62	−1.0
	L152	−2.36	−0.09	−2.46	−0.07	0.0	−0.07	0.14	−0.11	0.03	−2.78
AP9-CDK6/Vcyclin	I19	−2.36	−0.5	−2.86	−0.2	0.28	0.08	0.24	0.01	0.26	−2.95
	V27	−1.68	−0.16	−1.84	−0.12	0.15	0.03	0.09	−0.27	−0.18	−2.22
	A41	−0.73	−0.3	−1.03	0.07	−0.33	−0.26	−0.04	0.42	0.38	−0.98
	F98	−1.43	−0.15	−1.58	−0.12	0.22	0.1	0.02	−0.14	−0.12	−1.68
	V101	−1.0	−0.62	−1.62	−0.05	−2.65	−2.7	−0.01	1.7	1.69	−2.71
	Q103	−0.91	−0.76	−1.67	−0.18	−0.8	−0.98	0.25	0.51	0.76	−2.0
	L152	−2.51	−0.09	−2.6	−0.17	−0.2	−0.38	0.24	0.08	0.32	−2.95
AP9-CDK6	I19	−2.18	−0.39	−2.57	−0.23	0.2	−0.03	0.26	0.01	0.27	−2.76
	V27	−1.52	−0.15	−1.66	−0.12	0.16	0.03	0.11	−0.3	−0.18	−2.06
	A41	−0.62	−0.26	−0.88	0.07	−0.31	−0.24	−0.04	0.41	0.38	−0.81
	F98	−1.18	−0.17	−1.34	−0.12	0.23	0.11	0.12	−0.17	−0.05	−1.39
	V101	−0.93	−0.65	−1.58	−0.02	−2.35	−2.37	−0.04	1.7	1.66	−2.4
	Q103	−0.8	−0.69	−1.5	−0.15	−1.0	−1.15	0.2	0.69	0.88	−1.87
	L152	−2.38	−0.09	−2.47	−0.09	−0.2	−0.29	0.2	0.07	0.27	−2.82

^a^ All energy components are scaled in kcal/mol. $S_{vdW}$ and $B_{vdW}$, respectively, indicate contributions of the side chains and backbones to van der Waals interactions ($T_{vdW}$) of inhibitors with residues. $S_{ele}$ and $B_{ele}$, respectively, correspond to contributions of the side chains and backbones to electrostatic interactions ($T_{ele}$) of inhibitors with residues. $S_{gb}$ and $B_{gb}$, respectively, represent contributions of the side chains and backbones to inhibitor–residue polar solvation free energies.

**Table 3:** Hydrogen bonds formed between inhibitors and residues analyzed using CPPTRAJ.

Compound	^a^ Hydrogen Bonds	Distance (Å)	Angle (°)	^b^ Occupancy(%)
LQQ-CDK6/Vcyclin	V101-O…LQQ-N04-H4	2.8	153.5	98.8
	V101-N-H…LQQ-N01	3.2	147.7	77.7
	H100-NE2-HE2…LQQ-N05	3.0	155.0	65.4
	D163-N-H…LQQ-O01	3.2	151.2	53.0
LQQ-CDK6	V101-O…LQQ-N04-H4	2.8	153.4	98.7
	V101-N-H…LQQ-N01	3.2	148.0	79.7
	H100-NE2-HE2…LQQ-N05	3.0	155.3	58.8
	D163-N-H…LQQ-O01	3.1	151.1	51.6
AP9-CDK6/Vcyclin	V101-O…AP9-N6-H6	3.0	151.6	95.6
	V101-N-H…AP9-N7	3.3	138.1	59.7
AP9-CDK6	V101-O…AP9-N6-H6	3.0	154.3	91.6
	V101-N-H…AP9-N7	3.2	141.1	67.5

^a^ Hydrogen bonds are analyzed by an acceptor–donor distance of <3.5 Å and acceptor–H-donor angle of >120°. ^b^ Occupancy (%) is defined as the percentage of simulation time that a specific hydrogen bond exists.

## Data Availability

Data are contained within the article and Supplementary Materials.

## Supplementary Materials

The following supporting information can be downloaded at: https://www.mdpi.com/article/10.3390/molecules29112681/s1, Figure S1: Learning curves of the training and validation datasets. The metrics used here are loss and accuracy; Figure S2: The time course of RMSDs of inhibitors; Figure S3: The time course of RMSDs of proteins; Figure S4: The function of eigenvalues over eigenvector indexes; Table S1: Characteristic residue contacts (whose gradients from pixel attribution ≥0.7).
