# Supplementary material for: Unveiling Conformational States of CDK6 Caused by Binding of Vcyclin Protein and Inhibitor by Combining Gaussian Accelerated Molecular Dynamics and Deep Learning
Source: Molecules. 2024 Jun 5;29(11):2681. doi: 10.3390/molecules29112681 (PMC11174096; doi:10.3390/molecules29112681)
Supplement: Supplementary file 1 [file molecules-29-02681-s001.zip › molecules-3025128-supplementary.pdf]

Unveiling Conformational States of CDK6 Caused by Binding of Vcyclin Protein and Inhibitor by Combining Gaussian Accelerated Molecular Dynamics and Deep Learning

Lu Zhao *, Jian Wang, Wanchun Yang, Kunpeng Zhao, Qingtao Sun and Jianzhong Chen *

School of Science, Shandong Jiaotong University, Jinan 250357, China
* Correspondence: zhaolusdu@163.com (L.Z.); chenjianzhong1970@163.com or jzchen@sdjtu.edu.cn (J.C.)

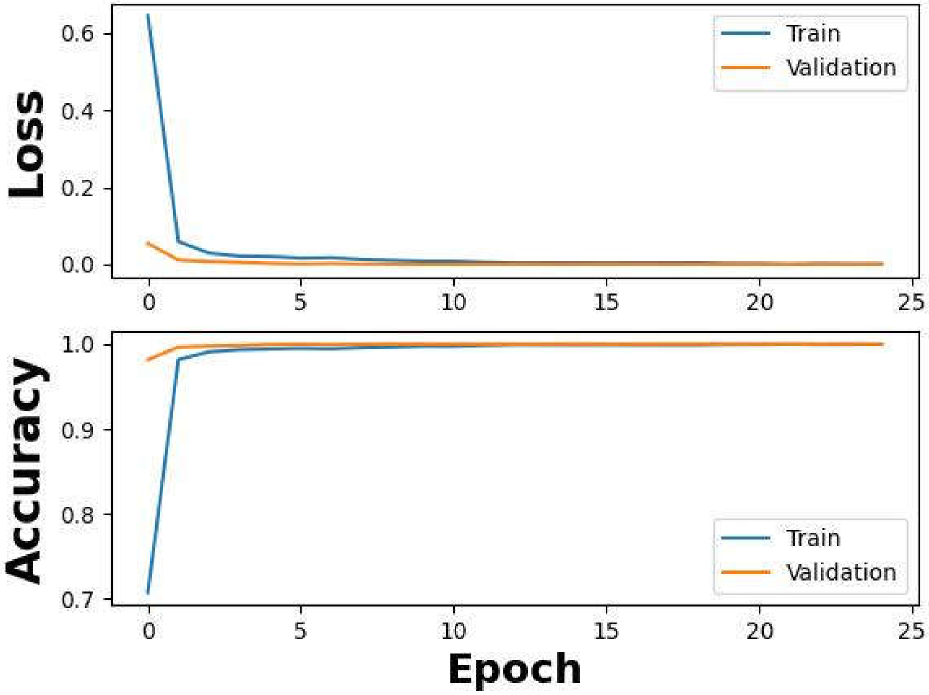

Figure S1. Learning curves of the training and validation datasets. The metrics used here are loss and accuracy.

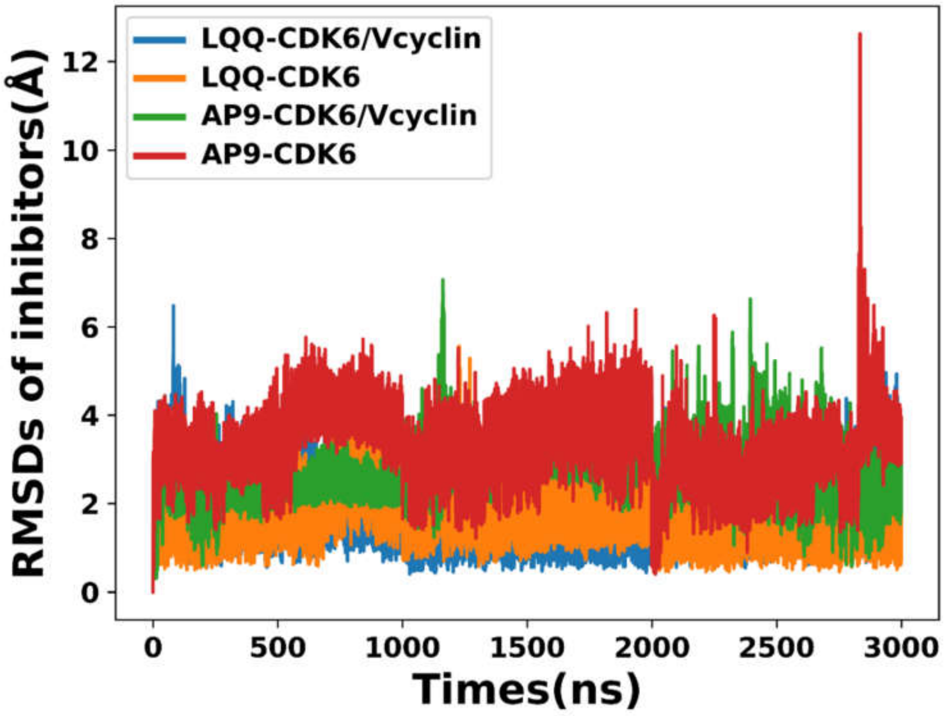

Figure S2. The time course of RMSDs of inhibitors.

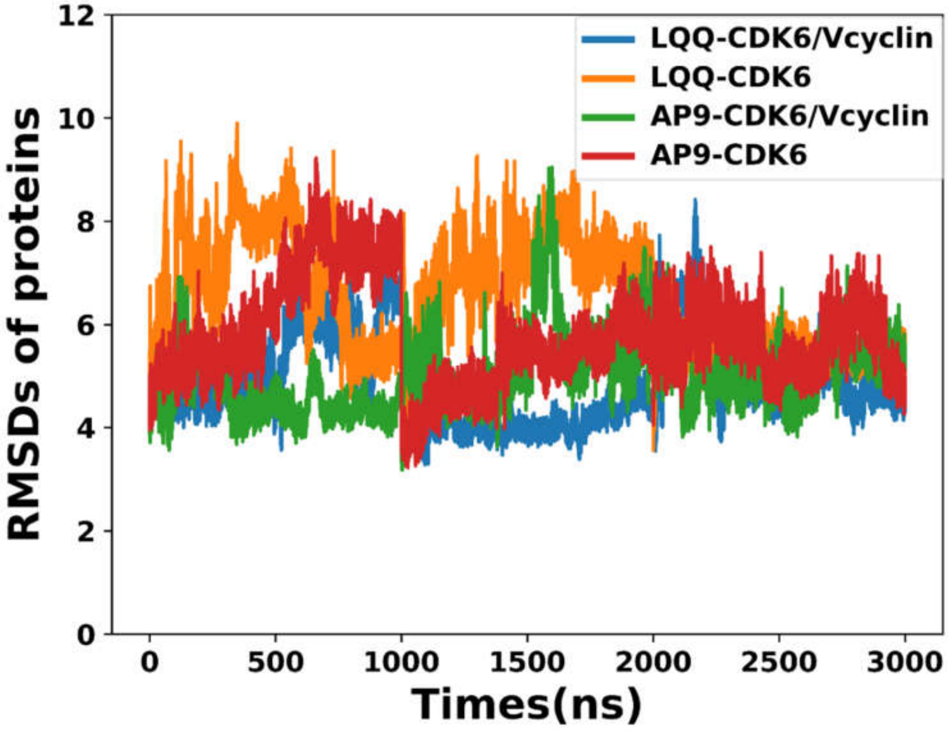

Figure S3. The time course of RMSDs of proteins.

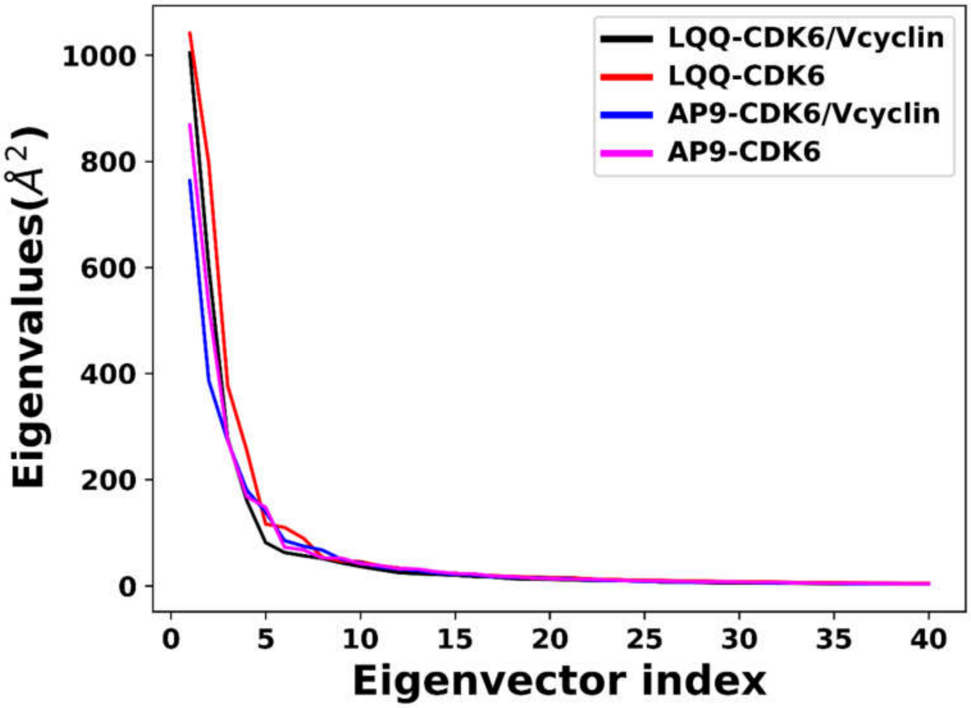

Figure S4. The function of eigenvalues over eigenvector indexes.

Table S1. Characteristic residue contacts (whose gradients from pixel attribution ⩾0.7).

Gradient	LQQ-bound CDK6/Vcyclin	LQQ-bound CDK6	AP9-bound CDK6/Vcyclin	AP9-bound CDK6
0.9	D163-G22	L166-A23	A162-E61	I59-M174<br>R60-A175<br>R60-T177
0.8	D163-A23			
0.7	G165-A23	G165-Y24<br>G165-A23		
